# How can audiovisual pathways enhance the temporal resolution of time-compressed speech in blind subjects?

**DOI:** 10.3389/fpsyg.2013.00530

**Published:** 2013-08-16

**Authors:** Ingo Hertrich, Susanne Dietrich, Hermann Ackermann

**Affiliations:** Department of General Neurology, Center of Neurology, Hertie Institute for Clinical Brain Research, University of TübingenTübingen, Germany

**Keywords:** speech perception, blindness, time-compressed speech, audiovisual pathways, speech timing

## Abstract

In blind people, the visual channel cannot assist face-to-face communication via lipreading or visual prosody. Nevertheless, the visual system may enhance the evaluation of auditory information due to its cross-links to (1) the auditory system, (2) supramodal representations, and (3) frontal action-related areas. Apart from feedback or top-down support of, for example, the processing of spatial or phonological representations, experimental data have shown that the visual system can impact auditory perception at more basic computational stages such as temporal signal resolution. For example, blind as compared to sighted subjects are more resistant against backward masking, and this ability appears to be associated with activity in visual cortex. Regarding the comprehension of continuous speech, blind subjects can learn to use accelerated text-to-speech systems for “reading” texts at ultra-fast speaking rates (>16 syllables/s), exceeding by far the normal range of 6 syllables/s. A functional magnetic resonance imaging study has shown that this ability, among other brain regions, significantly covaries with BOLD responses in bilateral pulvinar, right visual cortex, and left supplementary motor area. Furthermore, magnetoencephalographic measurements revealed a particular component in right occipital cortex phase-locked to the syllable onsets of accelerated speech. In sighted people, the “bottleneck” for understanding time-compressed speech seems related to higher demands for buffering phonological material and is, presumably, linked to frontal brain structures. On the other hand, the neurophysiological correlates of functions overcoming this bottleneck, seem to depend upon early visual cortex activity. The present Hypothesis and Theory paper outlines a model that aims at binding these data together, based on early cross-modal pathways that are already known from various audiovisual experiments on cross-modal adjustments during space, time, and object recognition.

## INTRODUCTION

Speech perception must be considered a multimodal process, arising as an audio-vibrational sensation even prior to birth (Spence and Decasper, 1987) and developing afterward into a primarily audiovisual event. Depending on environmental conditions, lip reading can significantly enhance speech perception (Sumby and Pollack, 1954; Ma et al., 2009). Within this context, the auditory and the visual data streams interact at different – functionally partially independent – computational levels as indicated by various psychophysical effects such as the McGurk and the ventriloquist phenomena (Bishop and Miller, 2011). Furthermore, in combination with cross-modal “equivalence representations” (Meltzoff and Moore, 1997) the visual channel supports early language acquisition, allowing for a direct imitation of mouth movements – based on an innate predisposition for the development of social communication (Streri et al., 2013). Presumably, the underlying mechanism relies on a general action recognition network that is known from primate studies (Buccino et al., 2004; Keysers and Fadiga, 2008), showing that action recognition is closely linked to the motor system, involving a variety of brain structures that have been summarized in a recent review (Molenberghs et al., 2012). In everyday life, the visual channel can be used, first, for the orientation of attention toward the speaking sound source, second, for lipreading, particularly in case of difficult acoustic environments and, third, for visual prosody providing the recipient with additional information related to several aspects of the communication process such as timing, emphasis, valence, or even semantic/pragmatic meaning of spoken language.

**FIGURE 1:**
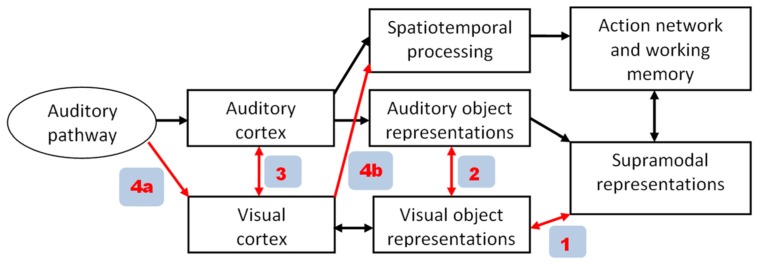
**Alternative pathways of visual cortex recruitment during auditory tasks in blind subjects.** In the model proposed in the present paper (see **Figure 2**), path 4a/4b plays a major role, enabling visual cortex to process event-timing based on afferent auditory information.

Given that speech perception encompasses audiovisual interactions, we must expect significant handicaps at least in early blind subjects with respect to spoken language capabilities. In line with this assumption, delayed speech acquisition has been observed in early blind children (Perez-Pereira and Conti-Ramsden, 1999). By contrast, however, various studies have shown that blind as compared to sighted individuals have superior abilities with respect to auditory perception, compensating at least partially for their visual deficits. Apart from altered central-auditory processing due to intra-modal neural plasticity in both early and late blind subjects (Elbert et al., 2002; Stevens and Weaver, 2009), blind individuals seem, furthermore, to use – at least some components – of their central visual system to support language-related representations (Röder et al., 2002). In principle, various pathways are available for visual cortex recruitment as shown in **Figure 1**. While particularly in early blind subjects backward projections from supramodal areas (red arrow #1 in **Figure 1**) seem to play a major role for visual cortex activation (Büchel, 2003), more direct pathways among secondary (#2) or primary sensory systems (#3) have also been postulated (Foxe and Schroeder, 2005). In the following we will provide some evidence that even afferent auditory information (#4a) can be utilized by the visual system in blind subjects. This information flow seems to refer to a timing aspect of event recording rather than object recognition (#4b).

Enhanced auditory processing in blind subjects appears to be associated with improved encoding of timing aspects of the acoustic signals. For example, congenitally blind individuals seem to preferentially pay attention to temporal as compared to spatial cues (Röder et al., 2007), and they outperform sighted subjects with respect to temporal resolution capabilities in psychoacoustic backward masking experiments (Stevens and Weaver, 2005). Furthermore, early as well as late blind subjects can acquire the ability to comprehend time-compressed speech at syllable rates up to ca. 20 syllables/s (normal range: ca. 4–8 syllables/s; Moos and Trouvain, 2007). During both backward masking experiments (Stevens et al., 2007) and ultra-fast speech perception (Hertrich et al., 2009, 2013; Dietrich et al., 2013), task performance-related activation of visual cortex has been observed. The aim of this Hypothesis and Theory paper is to delineate potential functional-neuroanatomic mechanisms engaged in enhanced perceptual processing of time-compressed speech in blind subjects. Since this ability has been observed in early as well as late blind individuals (Moos and Trouvain, 2007), we assume that the blind subjects rely on pathways also present in sighted people. However, these connections might not be available for ultra-fast speech processing in the latter group because they are engaged in the processing of actual visual signals.

Against the background of, first, functional magnetic resonance imaging (fMRI) and magnetoencephalographic (MEG) data recorded during the perception of time-compressed speech, second, the literature on cross-modal neuronal pathways in various species and, third, experimental findings dealing with audiovisual illusion effects, a model of visual cortex involvement in ultra-fast speech perception can be inferred. The issue of ultra-fast speech comprehension necessarily touches the question of a more general theory of continuous speech perception in the brain, including all subcomponents such as phonological encoding, lexical access, working memory, and sensorimotor activations of the articulatory system.

## NORMAL SPEECH PERCEPTION AND THE TEMPORAL BOTTLENECK

In principle, auditory cortex can follow the temporal envelope of verbal utterances across a wide range of speaking rates (Nourski et al., 2009), indicating that temporal resolution does not represent a limiting factor for the comprehension of time-compressed speech. Thus, we have to assume a “bottleneck” constraining the speed of spoken language encoding. Although the actual execution of motor programs is not required during speech perception, various studies have documented under these conditions the engagement of frontal areas associated with speech production (Pulvermüller et al., 2006). Furthermore, transcranial magnetic stimulation (TMS) experiments revealed these frontal activations to be functionally relevant, e.g., with respect to lexical processing (Kotz et al., 2010; D’Ausilio et al., 2012). Thus, any model of speech perception (e.g., Grimaldi, 2012) has to integrate action-related processing stages bound to the frontal lobe into the cerebral network leading from the acoustic signal to spoken language representations. These cortical areas, subserving, among other things, supramodal operations and transient memory functions, seem to be organized in a more or less parallel manner during speech and music perception (Patel, 2003).

A recent fMRI study (Vagharchakian et al., 2012) suggests that the “bottleneck” in sighted subjects for the comprehension of time-compressed speech arises from limited temporary storage capacities for phonological materials rather than speed constraints of the extraction of acoustic/phonetic features. As a consequence, phonological information might become “overwritten” before it can be fully encoded, a phenomenon contributing, presumably, to backward masking effects. The buffer mechanism for the comprehension of continuous speech has been attributed to left inferior frontal gyrus (IFG), anterior insula, precentral cortex, and upper frontal cortex including the supplementary motor area (SMA and pre-SMA; Vagharchakian et al., 2012). While IFG, anterior insula, and precentral gyrus are supposed to be bound to mechanisms of speech generation, pre-SMA and SMA might represent an important timing interface between perception- and action-related mechanisms, subserving, among other things, articulatory programming, inner speech, and working memory. More specifically, SMA has been assumed to trigger the execution of motor programs during the control of any motor activities, including speech production. For example, SMA is involved in the temporal organization and sequential performance of complex movement patterns (Tanji, 1994). This mesiofrontal area is closely connected to cortical and subcortical structures that adjust the time of movement initiation to a variety of internal and external demands. In case of acoustically cued simple motor tasks, SMA receives input from auditory cortex, as suggested by a study using Granger causality as a measure of connectivity (Abler et al., 2006). In case of more complex behavior requiring anticipatory synchronization of internal rhythms with external signals such as paced syllable repetitions, SMA seems to also play a major role both in the initiation and the maintenance of motor activity. Furthermore, there seem to be complementary interactions between SMA and the (upper right) cerebellum, the latter being particularly involved in case of increased demands on automation and processing speed during speech production (Riecker et al., 2005; Brendel et al., 2010).

Assuming visual cortex in blind individuals supports temporal signal resolution during speech perception, we have to specify, first, the trigger mechanisms of sighted subjects during perception of normal speech and, second, to delineate how the visual system engages in the encoding of temporal information. Concerning the former issue, Kotz et al. (2009) and Kotz and Schwartze (2010) put forward a comprehensive model of speech perception including an information channel that conveys auditory-prosodic temporal cues via subcortical pathways to pre-SMA and SMA proper. These suggestions also encompass the Asymmetric Sampling in Time hypothesis (Poeppel, 2003; Hickok and Poeppel, 2007) accounting for cortical hemisphere differences that are linked via reciprocal pathways to the cerebellum. As a major focus of the model referred to, Kotz and Schwartze (2010) tried to elucidate the relation of prosodic and syntactic processing – two functional subsystems that have to be coordinated. In analogy to prosody and syntax at the level of the sentence, the syllabic structure of speech, i.e., an aspect of prosody relevant to the timing and relative weighting of segmental phonetic information (Greenberg et al., 2003), provides a temporal grid for the generation of articulation-related speech representations in frontal cortex during perception. In line with the Asymmetric Sampling hypothesis, it has been shown that the syllabic amplitude modulation of the speech envelope is predominantly represented in the right hemisphere (Luo and Poeppel, 2007, 2012; Abrams et al., 2008). Against this background, we hypothesize that a right-hemisphere dominant syllabic timing mechanism is – somehow – linked via SMA to a left-dominant network of phonological processing during speech encoding.

The brain mechanisms combining low-frequency (theta band) syllabic and high-frequency (gamma band) segmental information have been outlined in a recent perspective paper (Giraud and Poeppel, 2012). This model must still be further specified with respect to, first, the pathways connecting right-hemisphere prosodic to left-hemisphere phonetic/phonological representations, second, the involved subcortical mechanisms and, third, the role of SMA for temporal coordination. Considering the salient functional role of syllabicity for speech comprehension (Greenberg et al., 2003), Giraud and Poeppel’s model can now be combined with a “syllabic” expansion of the prosodic subcortical-frontal mechanisms including SMA as outlined by Kotz et al. (2009) and Kotz and Schwartze (2010). In this expanded model, a syllable-based representation of speech within the frontal system of spoken language production is temporally coordinated with the incoming speech envelope.

Furthermore, close interactions between frontal speech generation mechanisms and permanent lexical representations have to be postulated since such interactions have also been shown to occur at the level of verbal working memory (Hickok and Poeppel, 2000; Buchsbaum and D’Esposito, 2008; Acheson et al., 2010). Although it must be assumed that verbal working memory, including articulatory loop mechanisms, is based on phonological output structures rather than the respective underlying lexical representations, recent data point at a continuous interaction between articulation-related phonological information and permanent lexical “word node” patterns (Romani et al., 2011). Furthermore, the permanent mental lexicon itself seems to have a dual structure that is linked to the ventral object recognition “what-” pathway within the anterior temporal lobe (phonological features and feature-based word forms; see De Witt and Rauschecker, 2012), on the one hand, and to the dorsal spatiotemporal and more action-related (“where-”) projections related to phonological gestures, on the other (Gow, 2012).

Concerning the comprehension of time-compressed speech, syllable rate appears to represent the critical limiting factor rather than missing phonetic information due to shortened segment durations, since insertion of regular silent intervals can largely improve intelligibility in normal subjects (Ghitza and Greenberg, 2009). Since, furthermore, the “bottleneck” seems to be associated with frontal cortex (Vagharchakian et al., 2012), it is tempting to assume that the lack of a syllable-prosodic representation at the level of the SMA limits the processing of time-compressed speech in case syllable rate exceeds a certain threshold. Auditory cortex can, in principle, track the envelope of ultra-fast speaking rates (Nourski et al., 2009) and even monitor considerably higher modulation frequencies, extending into the range of the fundamental frequency of a male speaking voice (Brugge et al., 2009; Hertrich et al., 2012). Furthermore, phase locking to amplitude modulations is consistently stronger within the right than the left hemisphere even at frequencies up to 110 Hz (Hertrich et al., 2004). However, the output from right auditory cortex might have a temporal limitation of syllabic/prosodic event recording: As soon as the modulation frequency approaches the audible range of pitch perception (ca. 16 Hz, that is, for example, the lowest note of an organ) prosodic event recording might compete with a representation of tonal structures. Furthermore, syllable duration at such high speaking rates (16 syllables/s, corresponding to a syllable duration of ca. 60 ms) may interfere with the temporal domain of phonetic features related to voice onset time or formant transitions (ca. 20–70 ms). Thus, the auditory system might not be able to track syllable onsets independently of the extraction of segmental phonological features. Although the segmental (left) and the prosodic (right) channels could be processed in different hemispheres, the timing of the two auditory cortices might be too tightly coupled in order to separate syllabic from segmental processing if the temporal domains overlap.

## A MODEL HOW VISUAL CORTEX IN BLIND SUBJECTS CAN ENHANCE THE PERCEPTION OF TIME-COMPRESSED SPEECH

**FIGURE 2:**
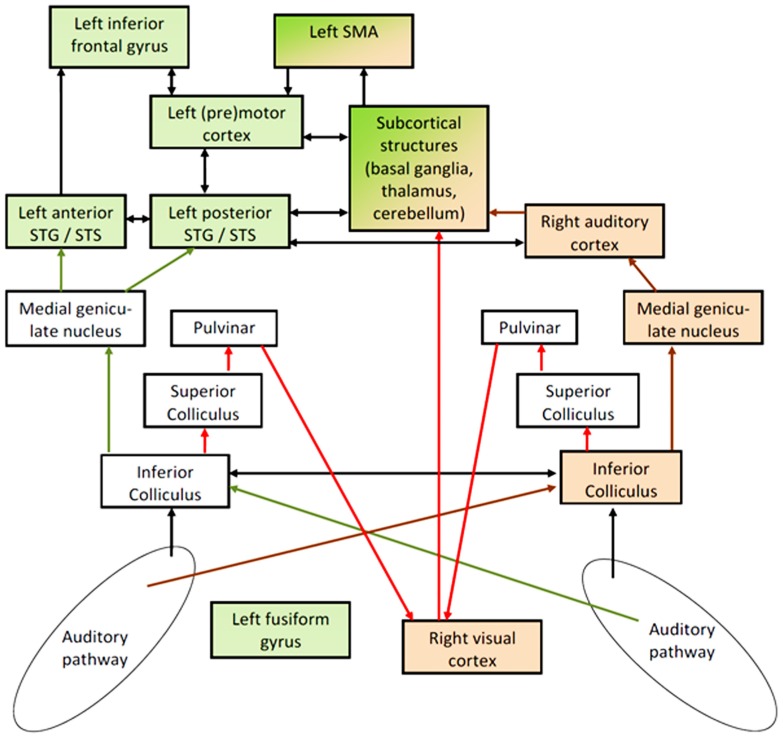
**Hypothetical pathways of speech perception: the phonological network – including secondary areas of left- hemisphere auditory cortex in superior temporal gyrus and sulcus (STG/STS) and frontal speech generation mechanisms – is colored in green, including additionally left fusiform gyrus (FG) in blind subjects.** This network seems to be linked to a right-dominant syllable-prosodic network via subcortical structures and supplementary motor area (SMA). In normal subjects, this prosodic network is mainly localized in the right-hemisphere auditory system (brown arrows). In order to overcome temporal constraints regarding this prosodic stream as an independent signal (independent from segmental processing and from pitch processing), blind subjects seem to be able to recruit part of their visual cortex – presumably via subcortical afferent auditory information (red arrows) – to represent this prosodic information and to transfer it as an event-trigger channel to the frontal part of the speech processing network. Arrows to and from left FG were omitted in order to avoid an overload of the model and since the major aspect addressed here is the interplay between the right-dominant prosodic and the left-dominant phonological network. Furthermore, direct pathways between visual and auditory cortex were also omitted since the “bottleneck” for understanding ultra-fast speech seems to be located in the interface between sensory processing and frontal speech generation mechanisms.

In this section, a model is presented suggesting right-hemisphere visual cortex activity to contribute to enhanced comprehension of ultra-fast speech in blind subjects. This model is supported, first, by the cortical activation patterns (fMRI, MEG) observed during spoken language understanding after vision loss (see Visual Cortex Involvement in Non-Visual Tasks) and, second, by studies dealing with early mechanisms of signal processing in the afferent audiovisual pathways (see Audiovisual Effects and Associated Pathways). Based, essentially, on the Asymmetric Sampling hypothesis (Poeppel, 2003; Hickok and Poeppel, 2007), the proposed model – as outlined in **Figure 2** – comprises two largely independent data streams, one representing phonological processing including auditory feature recognition in left superior temporal gyrus (STG), frontal speech generation mechanisms, and phonological working memory (green color). The other data stream provides a syllabic timing signal that, in sighted subjects, is predominantly represented at the level of the right-hemisphere auditory system (brown color). The SMA, presumably, synchronizes these two subsystems via subcortical structures (see Kotz and Schwartze, 2010). Blind subjects perceiving ultra-fast speech may use an alternative prosodic channel via an afferent audiovisual pathway including superior colliculus (SC), pulvinar (Pv), and right visual cortex (red arrows). In sighted subjects, these pathways contribute to auditory-driven gating and timing mechanisms for visual object recognition and/or are involved in visual mechanisms of spatial recalibration for auditory events. This afferent signal could provide the visual system with (meaningless) auditory temporal event markers. As a second step, the temporally marked visual events (in sighted) or “empty” visual events (in case of blind subjects) could be transferred to the frontal lobe for further processing such as the timing of inner speech and its encoding into working memory. In sighted subjects, the occipital-frontal pathways, among other things, contribute to the linkage of visually driven motor activity with the temporal structure of visual events.

Synchronization of the left-hemisphere phonological system with the incoming acoustic signal via a prosodic trigger mechanism – that, at an early stage, has some independence from the left-dominant pathway of phonological object recognition – appears to represent an important prerequisite for continuous speech perception under time-critical conditions. This prosodic timing channel, first, might trigger the extraction of phonological features by providing a syllabic grid since the phonological relevance and informational weight of phonological features depends on their position within a syllable (Greenberg et al., 2003). Presumably, transcallosal connections between right and left auditory cortex subserve these functions in sighted people. Second, the syllabic-prosodic timing signal could coordinate frontal speech generation and working memory mechanisms with the auditory input signal since speech generation is organized in a syllabic output structure. In particular, these interactions are important for the exact timing of top-down driven forward predictions with regard to the expected acoustic speech signal. Thus, the presence of a syllabic timing signal can significantly enhance the utilization of informational redundancy (predictability) during continuous realtime speech perception. It should also be mentioned that, although we assume an early signal-driven mechanism, visual cortex activation was found to be considerably weaker in case of (unintelligible) backward as compared to forward speech (Dietrich et al., 2013; Hertrich et al., 2013). We have to assume, thus, that top-down mechanisms providing information on the meaningfulness of the sound signal – arising, presumably, within frontal cortex – have an impact on the recruitment of the visual cortex during ultra-fast speech comprehension. Particularly, such interactions might be relevant for functional neuroplasticity processes during the training phase when blind subjects learn to accelerate their speech perception system using visual resources.

Apart from right-hemisphere mechanisms of prosody encoding, blind subjects seem also to engage ventral aspects (fusiform gyrus, FG) of their left-hemisphere visual system during ultra-fast speech perception (Hertrich et al., 2009; Dietrich et al., 2013). Therefore, left FG was added to **Figure 2** although the functional role of this occipito-temporal area remains to be further specified. At least parts of left FG appear to serve as a secondary phonological and/or visual word form area, linked to the left-hemisphere language processing network (McCandliss et al., 2003; Cao et al., 2008; Cone et al., 2008; Dietrich et al., 2013).

## VISUAL CORTEX INVOLVEMENT IN NON-VISUAL TASKS

A large number of studies report visual cortex activity in blind subjects during non-visual tasks, but the functional relevance of these observations is still a matter of debate (Röder et al., 2002; Burton, 2003; Burton et al., 2010; Kupers et al., 2011). Most studies (see Noppeney, 2007 for a comprehensive review) focus on early blind subjects, reporting visual cortex activity related to various tasks such as linguistic processing or braille reading. In some cases, a causal relationship has explicitly been demonstrated, e.g., by means of TMS showing that a transient “virtual lesion” in left occipital cortex interferes with semantic verbal processing (Amedi et al., 2004).

Regarding the neuronal mechanisms of functional cross-modal plasticity, cortico-cortical connections have been hypothesized on the basis of animal experiments, either direct cross-modal connections between, e.g., auditory and visual cortex, or backward projections from higher-order supramodal centers toward secondary and primary sensory areas (see e.g., Foxe and Schroeder, 2005; Bavelier and Hirshorn, 2010). Thereby, even in congenitally blind subjects, the supramodal representations seem to be quite similarly organized as in sighted individuals, indicating that supramodal representations form a stable pattern, largely independent of input modality (Ricciardi and Pietrini, 2011). In most examples of the engagement of the central visual system in blind subjects during non-visual cognitive tasks such as linguistic processing, thus, a top-down mode of stimulus processing from higher-order representations toward visual cortex has been assumed (Büchel et al., 1998; Büchel, 2003; Macaluso and Driver, 2005). By contrast, functional neuroplasticity via subcortical pathways has rarely been taken into account (Bavelier and Neville, 2002; Noppeney, 2007). As a phylogenetic example, blind mole rats, rodents with a largely inactive peripheral visual system, have developed an additional pathway conveying auditory input from inferior colliculus via dorsal lateral geniculate nucleus to the central visual system (Bronchti et al., 2002). In humans, however, this connection between the afferent auditory and the primary visual pathway does not seem to be implemented.

Our recent studies on blind subjects point to a further possibility of visual cortex involvement in an auditory task, i.e., listening to time-compressed speech. As a substitute for reading, blind individuals often use text-to-speech systems for the reception of texts. The speaking rate of these systems can be adjusted to quite high syllable rates, and blind users of these systems may learn to comprehend speech at rates up to ca. 20 syllables/s (Moos and Trouvain, 2007) while the normal speaking rate amounts to only 4–8 syllables/s. fMRI in blind subjects with the ability to understand ultra-fast speech at 16 syllables/s has shown hemodynamic activation, first, in left FG, a region that might be related to phonological representations (Cone et al., 2008) and, second, in right primary and secondary visual cortex, including parts of Brodmann areas (BA) 17 and 18 (Hertrich et al., 2009; Dietrich et al., 2013). Covariance analysis of fMRI data, furthermore, showed the ability to comprehend ultra-fast speech to be significantly associated, in addition to these two visual cortex areas, with activation in bilateral Pv, left IFG, left premotor cortex, left SMA as well as left anterior (aSTS) and bilateral posterior superior temporal sulcus (pSTS). As indicated by preliminary dynamic causal modeling (DCM) analyzes correlating functional connectivity with behavioral performance (Dietrich et al., 2010, 2011), the two visual areas activated in blind subjects, i.e., left-hemisphere FG and right-hemisphere primary and secondary visual cortex, seem to belong to different networks since they did not show significant connectivity in this analysis. FG, as part of the object-related ventral visual pathway (Haxby et al., 1991, 2000), might serve the representation of phonological “objects” linked to auditory and visual word form representations of the mental lexicon (McCandliss et al., 2003; Vigneau et al., 2006). Direct links between auditory and visual object representations have also been suggested to be activated by the use of sensory substitution devices “translating” optical signals into audible acoustic patterns (Striem-Amit et al., 2012). By contrast, right-dominant activation of early visual cortex as documented by Dietrich et al. (2013) seems to be associated with more elementary signal-related aspects as indicated by functional connectivity to pulvinar and auditory cortex. Furthermore, significant connectivity was observed between right visual cortex and left SMA, an area of temporal coordination in the frontal action network. Admittedly, considering the low temporal resolution of fMRI, this DCM analysis does not directly reflect the rapid information flow during speech perception. However, further evidence for an early signal-related rather than a higher-order linguistic aspect of speech processing being performed in right visual cortex has been provided by an MEG experiment (Hertrich et al., 2013). This study showed a particular signal component with a magnetic source in right occipital cortex that is phase-locked to a syllable onset signal derived from the speech envelope. The cross-correlation latency of this component was about 40–80 ms (see Figure 3 in Hertrich et al., 2013), indicating that this phase-locked activity arises quite early and, thus, might be driven by subcortical afferent input rather than cortico-cortical pathways. This might also be taken as an indicator that visual cortex activity represents a timing pattern rather than linguistic content. Thus, we hypothesize that visual cortex transfers a pre-linguistic prosodic signal, supporting the frontal action part of the speech perception network with timing information if the syllable rate exceeds the temporal resolution of the normal auditory prosody module. Admittedly, this model is still highly speculative given the limited basis of experimental data available so far. In addition, however, these suggestions shed some further light on exceptional abilities of blind subjects in the non-speech domain such as their resistance to backward masking as indicated by psychoacoustic experiments, pointing to a general mechanism of visual cortex recruitment for the purpose of time-critical event recording in blind subjects.

Taken together, left- and right-hemisphere activities observed in visual cortex of blind subjects during ultra-fast speech perception seem to be bound to the segmental (left) and prosodic (right) aspects of speech processing, in analogy to the Asymmetric Sampling hypothesis of the auditory system (Poeppel, 2003; Hickok and Poeppel, 2007). Activations of left-hemisphere phonological areas in the ventral visual stream can largely be expected on the basis of our knowledge regarding phonological and visual word form representations. By contrast, right visual cortex in blind subjects seems to belong to a different subsystem, receiving an afferent auditory timing signal that is related to syllable onsets and serving a similar function as the right-dominant prosodic timing channel in the theta band postulated for the auditory system (Abrams et al., 2008; Luo and Poeppel, 2012). However, the “prosodic” interpretation of right-hemisphere visual activities may require further support, first, with respect to existing pathways that could be able to build up such an extended prosodic network and, second, with respect to temporal resolution. Thus, in the following section various audiovisual experiments will be reviewed that can shed some light on the pathways contributing to visual system involvement in syllabic prosody representations.

## AUDIOVISUAL EFFECTS AND ASSOCIATED PATHWAYS

Very robust perceptual audiovisual interactions have been documented, such as the sound-induced multiple flash illusion. Irrespective of spatial disparity, these experiments have demonstrated that visual perception can be qualitatively altered by auditory input at an early level of processing. In case of this illusion, for example, a (physical) single flash is perceived as a double-flash if it is accompanied by a sequence of two short acoustic signals (Shams et al., 2000; Shams and Kim, 2010). The perception of the illusory second flash has been found to depend upon an early electrophysiological response component in the central visual system following the second sound at a latency of only 30–60 ms (Mishra et al., 2007). These experiments nicely show that the visual cortex is well able to capture acoustic event information at a high temporal resolution and at an early stage of processing. Further electrophysiological evidence for very fast audiovisual interactions has been obtained during simple reaction time tasks (Molholm et al., 2002).

Under natural conditions, early auditory-to-visual information transfer may serve to improve the detection of visual events although it seems to work in a quite unspecific manner with respect to both the location of the visual event in the visual field and cross-modal spatial congruence or incongruence (Fiebelkorn et al., 2011). Furthermore, spatially irrelevant sounds presented shortly before visual targets may speed up reaction times, even in the absence of any specific predictive value (Keetels and Vroomen, 2011). Such early audio-to-visual interactions seem to work predominantly as timing cues rather than signaling specific event-related attributes although some auditory spatial information can, in addition, be derived, e.g., when two data streams have to be segregated (Heron et al., 2012). Interestingly, the enhancement of visual target detection by auditory-to-visual information flow is not restricted to the actual event. Even passive repetitive auditory stimulation up to 30 min prior to a visual detection task can improve flash detection in the impaired hemifield of hemianopic patients (Lewald et al., 2012), indicating that auditory stimuli activate audiovisual pathways.

From a more general functional point of view, early audiovisual interactions facilitate the detection of cross-modal (in-)coherence of signals extending across both modalities. In this respect, there seems to be an asymmetry between the two channels with respect to temporal and spatial processing. In the temporal domain, the visual system appears to be adapted or gated (Purushothaman et al., 2012) by auditory information related to the time of acoustic signal onset (auditory dominance for timing). As a second step, the spatial representation of events within the dorsal auditory pathway may become recalibrated by coincident visual information (Wozny and Shams, 2011; spatial dominance of the visual system). This asymmetry, attributing temporal and spatial recalibration to different processing stages, can elucidate, for example, the differential interactions of these signal dimensions during the McGurk phenomenon (visual influence on auditory phonetic perception) as compared to the ventriloquist effect (visually induced spatial assignment of a speech signal to a speaking puppet Bishop and Miller, 2011). The McGurk effect is highly resistant against spatial incongruence, indicating an early binding mechanism (prior to the evaluation of spatial incongruence) on the basis of approximate temporal coincidence, followed by higher-order transfer of visual phonetic cues toward the auditory phonetic system. The temporal integration window of this effect has an asymmetrical structure and requires, as in natural stop consonant production, a temporal lag of the acoustic relative to the visual signal (Van Wassenhove et al., 2007). In this case, the visual component of the McGurk stimuli not only modifies, but also accelerates distinct electrophysiological responses such as the auditory-evoked N1 deflection (Van Wassenhove et al., 2005). However, an apparent motion design in which the shift between two pictures is exactly adjusted to the acoustic signal onset does not show such a visual effect on the auditory N1 response (Miki et al., 2004). In this latter case, presumably, early binding is not possible since the acoustic event trigger precedes the visual shift because of the delayed processing of actual visual signals. Thus, the McGurk effect seems to be based on a very early auditory-to-visual binding mechanism although its outcome might be the result of later higher-order phonological operations. By contrast, in case of the ventriloquist effect, the binding can be attributed to a later stage of spatial recalibration, top-down-driven by the perception of meaningful visual speech cues.

In contrast to syllabic event timing mechanisms assumed to engage visual cortex during ultra-fast speech perception, visuospatial cues are more or less irrelevant for blind subjects. The short latency (40–80 ms) of the MEG signal component phase-locked to syllable onsets over the right visual cortex (Hertrich et al., 2013) is comparable to the latency of visual cortex activity in case of the illusory double-flash perception, indicating a very early rather than late mechanism of visual cortex activation. As a consequence, we hypothesize that auditory timing information is derived from the acoustic signal at a pre-cortical stage, presumably, at the level of the SC, and then transferred to visual cortex via pulvinar and the posterior part of the secondary visual pathway. Although this pathway has been reported to target higher rather than primary visual areas (Martin, 2002; Berman and Wurtz, 2008, 2011), a diffusion tensor imaging tractography study indicates also the presence of connections from pulvinar to early cortical visual regions (Leh et al., 2008). As indicated by a monkey study, the pathway from pulvinar to V1 has a powerful gating function on visual cortex activity (Purushothaman et al., 2012). In sighted human subjects, the pulvinar-cortical visual pathway seems to play an important role with respect to Redundant Signal Effects (Maravita et al., 2008; see also Miller (1982) for behavioral effects of bimodal redundancy), multisensory spatial integration (Leo et al., 2008), audiovisual training of oculomotor functions during visual exploration (Passamonti et al., 2009), and suppression of visual motion effects during saccades (Berman and Wurtz, 2008, 2011). Regarding audiovisual interactions in sighted subjects such as the auditory-induced double-flash illusion (Shams et al., 2000; Mishra et al., 2007), the short latencies of electrophysiological responses of only 30–60 ms, by and large, rule out any significant impact of higher-order pathways from supramodal cortical regions to primary and secondary visual cortex as potential sources of this phenomenon, and even cross-modal cortico-cortical interactions between primary auditory and visual cortex might by too slow.

Cross-modal gating functions at the level of the auditory evoked P50, N100/M100 potentials as well as mismatch responses could be demonstrated within the framework of visual-to-auditory processing (Lebib et al., 2003; Van Wassenhove et al., 2005; Hertrich et al., 2007, 2009, 2011). Given that auditory event detection triggers visual event perception as in case of the auditory-induced double-flash illusion, it also seems possible that subcortical auditory information can trigger “visual” dummy events in the visual cortex of blind subjects. Subsequently, these event markers may function as a secondary temporal gating signal for the purpose of phonological encoding.

Frontal cortex, particularly, SMA, seems to play an important role in the coordination of phonological encoding with prosodic timing (see above). In principle, visual and audiovisual information via SC and pulvinar might reach frontal cortex in the absence of any activation of the occipital lobe (Liddell et al., 2005). However, this pathway is unlikely to be involved in the perception of ultra-fast speech since, first, it does not particularly involve SMA and, second, it is linked to reflexive action rather than conscious perception. Thus, we assume that in order to signalize an event-related trigger signal to the SMA, the data stream has to pass sensory cortical areas such somatosensory, auditory, or visual cortex. But how can audiovisual events (in sighted) or auditory-induced empty events represented in visual cortex (in blind people) feed timing information into SMA? A comprehensive study of the efferent and afferent connections of this mesiofrontal area in squirrel monkeys found multiple cortical and subcortical pathways, but no direct input from primary or secondary visual cortex. By contrast, proprioception, probably due to its close relationship to motor control, seems to have a more direct influence on SMA activity (Jürgens, 1984). Regarding the visual domain, SMA seems to be involved in visually cued motor tasks (Mohamed et al., 2003) and in visually guided tracking tasks (Picard and Strick, 2003) as well as in an interaction of visual event detection with oral conversation as shown by reaction time effects (Bowyer et al., 2009). Thus, in analogy to the auditory models of Hickok and Poeppel (2007) and Kotz and Schwartze (2010), we may assume a pathway from the right-hemisphere dorsal visual stream, representing syllabic events, toward the SMA via subcortical structures including the thalamus and the (left) cerebellum.

## DISCUSSION

In summary, the present model assumes a dual data stream to support the linguistic encoding of continuous speech: predominant left-hemisphere extraction of phonetic features and predominant right-hemisphere capture of the speech envelope. The coordination of these two functional subsystems seems to be bound to the frontal cortex. More specifically, SMA might critically contribute to the synchronization of the incoming signal with top-down driven syllabically organized sequential pacing signals. In case of ultra-fast speech, the auditory system – although capable to process signals within the 16 Hz domain – may fail to separate syllable-prosodic and segmental information at such high rates. Therefore, the speech generation system, including the phonological working memory, cannot be triggered by a prosodic event channel. In order to overcome this bottleneck, we must either learn to encode speech signals in the absence of a syllabic channel – a, most presumably, quite difficult task – or we have to recruit a further neural pathway to provide the frontal cortex with syllabic information. The latter strategy seems to be available to blind subjects who may use the audiovisual interface of the secondary visual pathway in order to transmit syllabic event triggers via pulvinar to right visual cortex. As a consequence, the tentative function of visual cortex might consist in the transformation of the received timing signal into a series of (syllabic) events that subsequently can be conveyed to the frontal lobe in order to trigger the phonological representations in the speech generation and working memory system. These “events” might be similar to the ones that, in sighted subjects, become spatially recalibrated by vision. Since vision loss precludes any spatial recalibration, the auditory events may target a region near the center of the retinotopic area in visual cortex. Considering, first, that this audiovisual pathway is linked to visuospatial processing in sighted subjects and, second, that the extracted auditory signal components are prosodic event-related rather than phonological data structures, it seems rather natural that they are preferably processed within the right-hemisphere. Thus, by “outsourcing” the syllabic channel into the visual system, blind people may overcome the prosodic event timing limits of right-hemisphere auditory cortex.

Various aspects of the proposed model must now be tested explicitly, e.g., by means of TMS techniques and further connectivity analyzes. Assuming, for example, that right visual cortex of blind subjects is involved in prosodic timing mechanisms, a virtual lesion of this area during ultra-fast speech perception must be expected to yield similar comprehension deficits as virtual damage to right auditory cortex in sighted subjects during perception of moderately fast speech. Furthermore, pre-activation of right visual cortex as well as co-activation of right visual cortex with SMA might have facilitating effects on speech processing. In sighted subjects, furthermore, it should be possible to simulate the early phase-locked activity in right visual cortex by presenting flashes that are synchronized with syllable rate. If, indeed, visual cortex can forward prosodic event triggers, these flashes should enhance the comprehension of time-compressed speech.

So far, only few studies provide clear-cut evidence for a subcortical audiovisual pathway targeting primary visual cortex. The present model postulates that a speech envelope signal is already represented at a pre-cortical level of the brain. As a consequence, the prosodic timing channel engaged in speech processing should be separated from the “segmental” auditory channel already at a subcortical stage. So far, recordings of brainstem potentials did not reveal any lateralization effects similar to the cortical distinction of short-term segmental (left hemisphere) and low-frequency suprasegmental/prosodic (right-hemisphere) information (Abrams et al., 2010). At the level of the thalamus, however, low-frequency information is well represented, and it has been hypothesized that these signals – bound predominantly to paralemniscal pathways – have a gating function regarding the perceptual evaluation of auditory events (He, 2003; Abrams et al., 2011). Furthermore, the underlying temporal coding mechanism (spike timing) seems to be particularly involved in the processing of communication sounds via thalamus, primary and non-primary auditory cortex up to frontal areas (Huetz et al., 2011).

Alternatively, one might suggest that the visual cortex of blind individuals is activated by cross-modal cortico-cortical pathways. In sighted subjects, however, early audiovisual interactions allowing for the enhancement of auditory processing by visual cues require a time-lead of the visual channel extending from 20 to 80 ms (Kayser et al., 2008). Thus, it seems implausible that ultra-fast speech comprehension can be accelerated by visual cortex activation via cortico-cortical cross-modal pathways. If the visual channel is really capable to impact auditory encoding of speech signals at an early phase-locked stage, then very early subcortical afferent input to the visual system must be postulated. These fast connections might trigger phonological encoding in a manner analogous to the prosodic timing mechanisms in right-hemisphere auditory cortex. The underlying mechanism of this process might consist in phase modulation of oscillatory activity within visual cortex based on subcortical representations of the speech envelope.

Since the “bottleneck” for understanding ultra-fast speech in sighted subjects has been assigned to frontal rather than temporal regions, pathways projecting from visual to frontal cortex, targeting, in particular, SMA, must be assumed in order to understand how blind people can overcome these constraints. The connections sighted subjects use to control the motor system during visual perception, both in association with ocular and visually guided upper limb movements, represent a plausible candidate structure. Considering SMA a motor timing device with multiple input channels but no direct interconnections with primary visual cortex, the transfer of the prosodic signals toward SMA might be performed via subcortical mechanisms involving cerebellum, basal ganglia, and thalamus. However, in upcoming studies this has to be demonstrated explicitly.

The present model might also contribute to a better understanding of previous findings on enhanced auditory performance of blind individuals such as resistance to backward masking, as documented by Stevens and Weaver (2005). Thereby, this aspect of temporal processing seems to be related to perceptual consolidation rather than elementary auditory time resolution. Furthermore, resistance to backward masking in blind subjects was associated with activity, even preparatory activity in visual cortex. In line with the present model, activation of visual cortex was found in the right rather than the left hemisphere. Stevens et al. (2007) interpreted the preparatory visual activation as a “baseline shift” related to attentional modulation. However, they did not provide an explicit hypothesis about the nature of the input signal toward visual cortex. Based on the present model, we might assume that the secondary visual pathway provides the visual system with afferent auditory information. Considering brain activations outside the visual system, Stevens et al. (2007) did not mention SMA, but other frontal regions such as the frontal eye field, known as a structure serving auditory attentional processing in blind subjects (Garg et al., 2007). Thus, at least some aspects of the present model might be expanded to the non-speech domain, referring to a general mechanism that enhances the temporal resolution of auditory event recording by using the afferent audiovisual interface toward the secondary visual pathway.

At least partially, the assumption of an early signal-related transfer mechanism via pulvinar, secondary visual pathway, and right visual cortex toward the frontal cortex was based on fMRI connectivity analyzes, an approach of still limited temporal resolution. So far, it cannot be excluded that frontal cortex activation under these conditions simply might reflect higher-order linguistic processes that are secondary to, but not necessary for comprehension. Nevertheless, functional imaging data revealed the time constraints of speech understanding to be associated with frontal structures (Vagharchakian et al., 2012). Thus, frontal lobe activity during spoken language comprehension seems comprise both the generation of inner speech after lexical access and the generation of well-timed predictions regarding the syllabically organized structure of upcoming speech material. In other words, it is an interface between bottom-up and top-down mechanisms.

## Conflict of Interest Statement

The authors declare that the research was conducted in the absence of any commercial or financial relationships that could be construed as a potential conflict of interest.
